# Supplementary material for: Impact of extracorporeal membrane oxygenation-related complications on in-hospital mortality
Source: PLoS One. 2024 Mar 25;19(3):e0300713. doi: 10.1371/journal.pone.0300713 (PMC10962856; doi:10.1371/journal.pone.0300713)

S1 Fig. Schematic representative figures of VA and VV ECMO. VA, venoarterial; VV, venovenous; ECMO, extracorporeal membrane oxygenation.

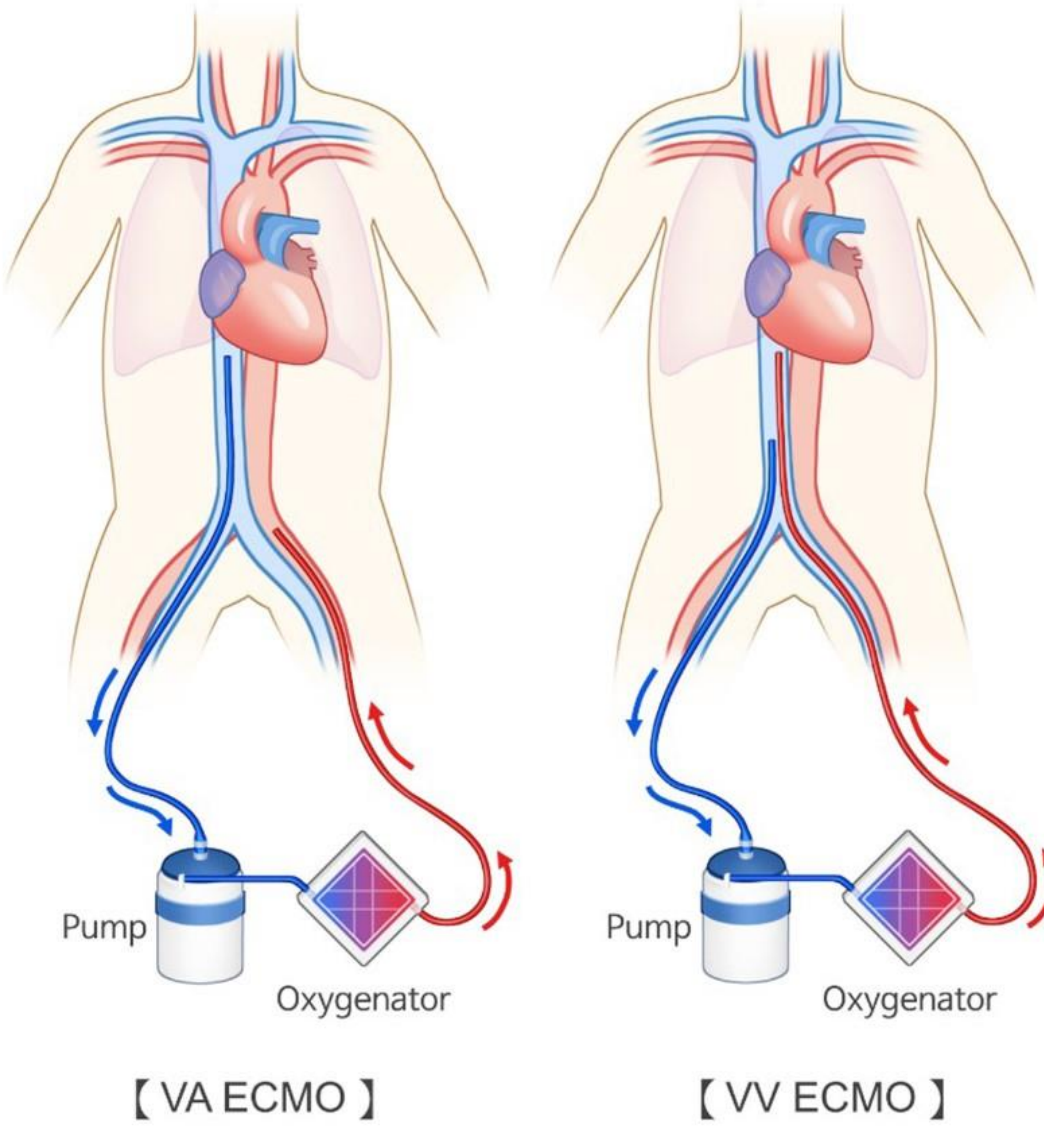

Supplement: S1 Fig — (PDF) [file pone.0300713.s001.pdf]
