# Supplementary material for: Impact of extracorporeal membrane oxygenation-related complications on in-hospital mortality
Source: PLoS One. 2024 Mar 25;19(3):e0300713. doi: 10.1371/journal.pone.0300713 (PMC10962856; doi:10.1371/journal.pone.0300713)

S2 Fig. Flow chart of ECMO inclusion. The analysis included 856 ECMO runs from 769 patients who underwent ECMO. Eligible ECMO procedures were stratified into 2 groups according to the type of ECMO: VA ECMO (*n* = 709, 82.8%) and VV ECMO (*n* = 147, 17.2%). ECMO, extracorporeal membrane oxygenation; VA, venoarterial; VV, venovenous.

*Nine patients underwent both VA and VV ECMO runs and were included in both the VA and VV ECMO groups.

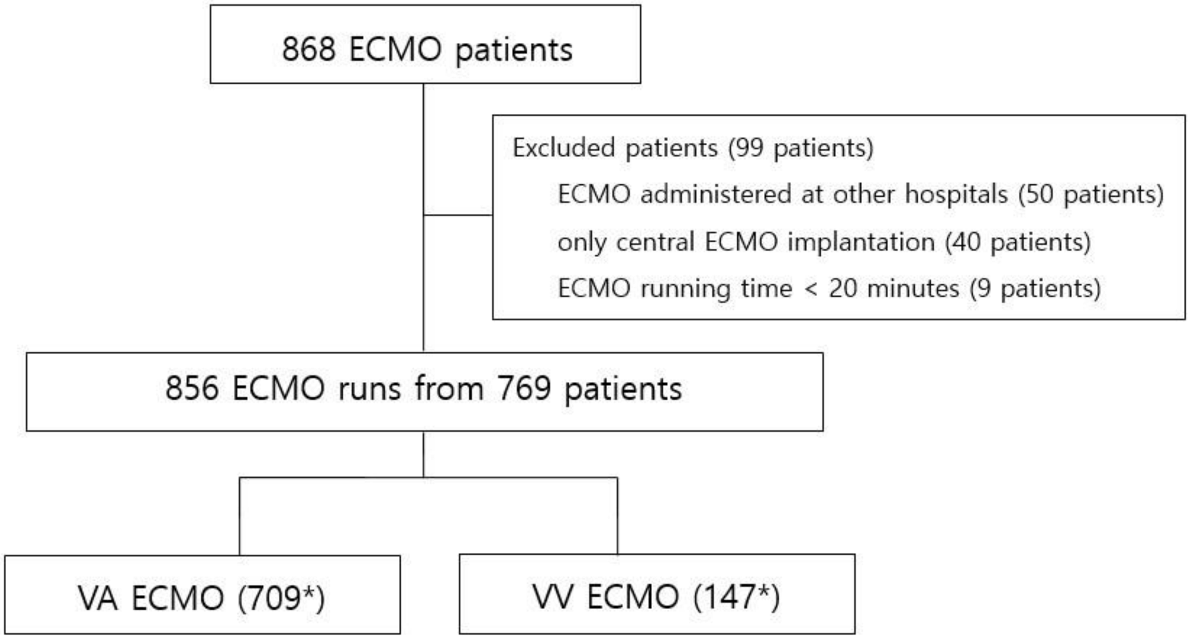

Supplement: S2 Fig — (PDF) [file pone.0300713.s002.pdf]
