# Supplementary material for: Impact of extracorporeal membrane oxygenation-related complications on in-hospital mortality
Source: PLoS One. 2024 Mar 25;19(3):e0300713. doi: 10.1371/journal.pone.0300713 (PMC10962856; doi:10.1371/journal.pone.0300713)
Supplement: S1 Text — (PDF) [file pone.0300713.s003.pdf]

S1 Methods

Extracorporeal membrane oxygenation (ECMO) protocol

ECMO was administered using a standard protocol [1–5]. Because of the heterogeneity of our study sample, there were no predefined indications for ECMO support. The decision to administer ECMO was made by each physician but usually followed general recommendations in the Extracorporeal Life Support Organization (ELSO) guidelines [4,5]. In most, if not all, cases, the cannulation was performed using a percutaneous technique, preferably guided by ultrasonography performed by specialized cardiac surgeons with expertise in both percutaneous and open procedures. If percutaneous cannulation failed, direct cut-down cannulation was done. The heparin bolus was given at any time after the main wire was placed. Two ECMO systems were used—the Quadrox PLS System (Maquet Cardiopulmonary AG, Rastatt, Germany) and the Capiox EBS System (Terumo Cardiovascular Systems Corp., Tokyo, Japan)—with each system having its own oxygenator, pump, and console. Most patients were anticoagulated with unfractionated heparin, except those who had contraindications for heparinization. The target range of activated clotting time and activated partial thromboplastin time on ECMO were 150 to 180 and 60 to 80 s, respectively. ECMO weaning could be considered if the level of oxygen or carbon dioxide was adequate without any evidence of low cardiac output after 1 to 2 h of interruption of gas flow in venovenous ECMO or with low ECMO flow (<1.5 L/min), low gas flow (<2.0 L/min), and ECMO FiO_2 30 to 40%, and after 5 min of pump controlled retrograde trial off in venoarterial ECMO.

References

[1] Ellouze O, Abbad X, Constandache T, Missaoui A, Berthoud V, Daily T, et al. Risk Factors of Bleeding in Patients Undergoing Venoarterial Extracorporeal Membrane Oxygenation. Ann Thorac Surg 2021;111:623–628.

[2] Parzy G, Daviet F, Persico N, Rambaud R, Scemama U, Adda M, et al. Prevalence and Risk Factors for Thrombotic Complications Following Venovenous Extracorporeal Membrane Oxygenation: A CT Scan Study. Crit Care Med 2020;48:192–199.

[3] Tonna JE, Abrams D, Brodie D, Greenwood JC, Rubio Mateo-Sidron JA, Usman A, et al. Management of Adult Patients Supported with Venovenous Extracorporeal Membrane Oxygenation (VV ECMO): Guideline from the Extracorporeal Life Support Organization (ELSO). ASAIO J 2021;67:601–610.

[4] Richardson ASC, Tonna JE, Nanjayya V, Nixon P, Abrams DC, Raman L, et al.. Extracorporeal Cardiopulmonary Resuscitation in Adults. Interim Guideline Consensus Statement From the Extracorporeal Life Support Organization. ASAIO J 2021;67:221–228.

[5] Extracorporeal Life Support Organization (ELSO): ELSO guidelines for cardiopulmonary extracorporeal life support, Version 1.4 August 2017, Ann Arbor, MI. Available at: http://www.elso.org. Accessed April 23, 2021.

Hb, hemoglobin; ref., reference range; PLT, platelet.
