# Supplementary material for: Impact of extracorporeal membrane oxygenation-related complications on in-hospital mortality
Source: PLoS One. 2024 Mar 25;19(3):e0300713. doi: 10.1371/journal.pone.0300713 (PMC10962856; doi:10.1371/journal.pone.0300713)
Supplement: S1 Table — (PDF) [file pone.0300713.s004.pdf]

S1 Table. Baseline and clinical characteristics, stratified by the ECMO mode.

Per-patient basis	**Total**	**VA ECMO**[a]	**VV ECMO**[a]	***P*-value**
**Number of patients**	769	639	139	
**Mean age, years ± SD**	59.4 ± 13.8	59.8 ± 14.1	57.1 ± 12.5	0.04
**Male**	246 (32.0)	199 (31.1)[b]	50 (36.0)	0.32
**BMI, kg/m^2**	23.5 ± 4.5	23.6 ± 4.4	23.0 ± 5.0	0.18
**Risk factor**				
Hypertension	336 (43.7)	291 (45.5)	48 (34.5)	0.02
Diabetes mellitus	219 (28.5)	194 (30.4)	26 (18.7)	0.01
Smoking	181 (23.5)	153 (23.9)	30 (21.6)	0.63
PAOD[c]	19 (2.5)	17 (2.7)	2 (1.4)	0.55
**Medical history**[d]				
History of CAD	164 (21.3)	157 (24.6)	7 (5.0)	<0.01
History of CVA	164 (21.3)	54 (8.5)	3 (2.2)	0.01
History of CKD	108 (14.0)	97 (15.2)	12 (8.6)	0.06
Use of antiplatelet	199 (25.9)	186 (29.1)	14 (10.1)	<0.01
Use of anticoagulant	109 (14.2)	104 (16.3)	7 (5.0)	<0.01
Per-ECMO basis	Total	VA ECMO	VV ECMO	*P*-value
**Number of ECMO runs**	856	709 (82.8)	147 (17.2)	
**Indications**				<0.01
Cardiac failure	489 (57.1)	487 (68.7)	2 (1.4)	
Post-cardiotomy shock	148 (17.3)	144 (20.3)	4 (2.7)	
Respiratory failure	184 (21.5)	57 (8.0)	127 (86.4)	
PTE	13 (1.5)	13 (1.8)	0 (0.0)	
Septic shock	9 (1.1)	8 (1.1)	1 (0.7)	
Others	13 (1.5)	0 (0.0)	13 (8.8)	
**CPCR**[e]	313 (36.6)	304 (42.9)	9 (6.1)	<0.01
**CRRT**[e]	543 (63.4)	487 (68.7)	56 (38.1)	<0.01
**Arterial cannulation**				
Cannula size, Fr. median (IQR)-	-	15 (15–17)		
Distal perfusion catheter	-	224 (31.6)		

Artery closure				
Surgical repair	-	108 (15.2)		
Perclosure	-	50 (7.1)		
**Venous cannulation**				
Cannula size, out, Fr. median (IQR)	-		22 (21–23)	
Cannula size, in, Fr. median (IQR)	-		18 (17–19)	
**Success of ECMO weaning**	461 (53.9)	366 (51.6)	95 (64.6)	<0.01
Operation	104 (12.1)	79 (11.1)	25 (17.0)	
**Hospital stay, days**	56.7 ± 96.6	51.7 ± 79.4	80.6 ± 153.0	<0.01
ICU stay, days	28.2 ± 39.4	26.0 ± 37.2	38.9 ± 47.4	<0.01
**In-hospital death**	417 (48.7)	374 (52.8)	43 (29.3)	<0.01
**Cause of death**				
ECMO-related[f]	24 (5.8)	23 (6.1)	1 (2.3)	0.49
Cardiac failure	189 (45.3)	186 (49.7)	3 (7.0)	<0.01
Respiratory failure	96 (23.0)	64 (17.1)	32 (74.4)	<0.01
Septic shock	43 (10.3)	39 (10.4)	4 (9.3)	>0.99
Cerebrovascular disease	6 (1.4)	5 (1.3)	1 (2.3)	0.48
Bleeding	37 (8.9)	35 (9.4)	2 (4.7)	0.41
Others	22 (5.3)	22 (5.9)	0 (0.0)	0.15

Categorical data are given as numbers (%); continuous data are presented as mean ± SD or median (IQR). ECMO, extracorporeal membrane oxygenation; VA, venoarterial; VV, venovenous; SD, standard deviation; BMI, body mass index; PAOD, peripheral arterial occlusive disease; CAD, coronary artery disease; CVA, cerebrovascular accident; CKD, chronic kidney disease; PTE, pulmonary thromboembolism; CPCR, cardiopulmonary cerebral resuscitation; CRRT, continuous renal replacement therapy; IQR, interquartile range; ICU, intensive care unit.

[a] Included 9 patients who underwent both VA and VV ECMO runs.

[b] Included 3 men who underwent both VA and VV ECMO runs.

[c] PAOD was defined as a history of surgical or radiologic intervention or an ankle–brachial index ≤ 0.9 prior to ECMO implantation.

[d] Medical history prior to ECMO implantation.

[e] CPCR or CRRT prior to ECMO implantation.

[f] Included ECMO-related vascular and cerebrovascular complications.
