# Supplementary material for: Impact of extracorporeal membrane oxygenation-related complications on in-hospital mortality
Source: PLoS One. 2024 Mar 25;19(3):e0300713. doi: 10.1371/journal.pone.0300713 (PMC10962856; doi:10.1371/journal.pone.0300713)
Supplement: S2 Table — (PDF) [file pone.0300713.s005.pdf]

S2 Table. In-hospital mortality rates according to ECMO indications.

	No. of ECMO runs	VA ECMO	VV ECMO
**Cardiac failure**	285/489 (58.3)	285/487 (53.0)	0/2
**Post-cardiotomy shock**	66/148 (44.6)	66/144 (45.8)	0/4
**Respiratory failure**	77/184 (41.8)	36/57 (63.2)	41/127 (32.3)
**PTE**	7/13 (53.8)	7/13 (53.8)	0/0
**Septic shock**	7/9 (77.8)	7/8 (87.5)	0/1
**Others**	2/13 (29.3)	0/0	2/13 (29.3)

Values in parentheses are percentages.

ECMO, extracorporeal membrane oxygenation; VA, venoarterial; VV, venovenous; PTE, pulmonary thromboembolism.
