# Supplementary material for: Impact of extracorporeal membrane oxygenation-related complications on in-hospital mortality
Source: PLoS One. 2024 Mar 25;19(3):e0300713. doi: 10.1371/journal.pone.0300713 (PMC10962856; doi:10.1371/journal.pone.0300713)
Supplement: S3 Table — (PDF) [file pone.0300713.s006.pdf]

S3 Table. Factors associated with ECMO-related thromboembolic complications in VA ECMO.

	Univariable analysis OR (95% CI)	*P*-value	Multivariable analysis OR (95% CI)	*P*-value
**Age**	0.97 (0.95–0.99)	<0.01	0.98 (0.95–1.00)	0.02
**Female**	1.63 (0.85–3.11)	0.14	NA	NA
**BMI**	0.95 (0.87–1.03)	0.22	NA	NA
**Hypertension**	0.64 (0.33–1.23)	0.18	NA	NA
**Diabetes mellitus**	1.02 (0.52–2.02)	0.95	NA	NA
**Smoking**	1.64 (0.84–3.21)	0.15	NA	NA
**PAOD**	0.97 (0.12–7.53)	0.98	NA	NA
**History of CAD**	0.70 (0.32–1.55)	0.38	NA	NA
**History of CVA**	1.08 (0.36–3.24)	0.89	NA	NA
**History of CKD**	1.51 (0.68–3.38)	0.31	NA	NA
**CPCR**	0.54 (0.22–1.33)	0.12	NA	NA
**CRRT**	5.74 (1.95–16.87)	<0.01	5.27 (1.66–16.77)	<0.01
**ECMO running time (10 h)**	1.01 (1.00–1.01)		1.00 (0.99–1.01)	0.46
**Arterial cannula size**	0.99 (0.77–1.27)		NA	NA
**Initial Hb (ref. ≥ 10.0 g/dL)**		0.26		
<8.0 g/dL	3.10 (0.74–13.04)	0.12	NA	NA
8.0–10.0 g/dL	1.73 (0.55–5.48)	0.35	NA	NA
**Initial PLT (ref. ≥ 100×10^3/μL)**		0.04		0.25
<50×10^3/μL	3.46 (1.35–8.89)	0.01	2.27 (0.81–6.38)	0.12
50–100(×10^3)/μL	1.07 (0.32–3.63)	0.91	0.58 (0.09–3.84)	0.57

ECMO, extracorporeal membrane oxygenation; VA, venoarterial; OR, odds ratio; CI, confidence interval; BMI, body mass index; NA, not applicable; PAOD, peripheral arterial occlusive disease; CAD, coronary artery disease; CVA, cerebrovascular accident; CKD, chronic kidney disease; CPCR, cardiopulmonary cerebral resuscitation; CRRT, continuous renal replacement therapy; Hb, hemoglobin; ref., reference range; PLT, platelet.
