# Supplementary material for: Impact of extracorporeal membrane oxygenation-related complications on in-hospital mortality
Source: PLoS One. 2024 Mar 25;19(3):e0300713. doi: 10.1371/journal.pone.0300713 (PMC10962856; doi:10.1371/journal.pone.0300713)
Supplement: S4 Table — (PDF) [file pone.0300713.s007.pdf]

S4 Table. Factors associated with ECMO-related bleeding complications in VA ECMO.

	Univariable analysis OR (95% CI)	*P*-value	Multivariable analysis OR (95% CI)	*P*-value
**Age**	1.00 (0.99–1.02)	0.83	NA	NA
**Female**	1.25 (0.72–2.16)	0.43	NA	NA
**BMI**	0.95 (0.89–1.00)	0.054	NA	NA
**Hypertension**	0.68 (0.40–1.17)	0.16	NA	NA
**Diabetes mellitus**	0.73 (0.40–1.35)	0.32	NA	NA
**Smoking**	0.74 (0.39–1.39)	0.35	NA	NA
**PAOD**	1.21 (0.27–5.44)	0.80	NA	NA
**History of CAD**	0.73 (0.37–1.41)	0.34	NA	NA
**History of CVA**	1.19 (0.44–3.19)	0.73	NA	NA
**History of CKD**	1.51 (0.78–2.90)	0.22	NA	NA
**CPCR**	1.19 (0.72–1.98)	0.50	NA	NA
**CRRT**	1.74 (0.94–3.22)	0.08	NA	NA
**ECMO running time (10 h)**	1.01 (1.00–1.02)	<0.01	1.01 (1.00–1.02)	<0.01
**Arterial cannula size**	0.79 (0.64–0.96)	0.02	0.79 (0.63–0.99)	0.04
**Initial Hb (ref. ≥ 10.0 g/dL)**		<0.01		<0.01
<8.0 g/dL	5.29 (2.84–9.86)	<0.01	4.19 (2.21–7.96)	<0.01
8.0–10.0 g/dL	1.15 (0.62–2.13)	0.66	0.90 (0.46–1.74)	0.75
**Initial PLT (ref. ≥ 100×10^3/μL)**		<0.01		0.06
<50×10^3/μL	2.21 (1.03–4.74)	0.04	0.90 (0.46–1.74)	0.35
50–100(×10^3)/μL	2.61 (1.50–4.55)	<0.01	2.07 (1.13–3.79)	0.02

ECMO, extracorporeal membrane oxygenation; VA, venoarterial; OR, odds ratio; CI, confidence interval; BMI, body mass index; NA, not applicable; PAOD, peripheral arterial occlusive disease; CAD, coronary artery disease; CVA, cerebrovascular accident; CKD, chronic kidney disease; CPCR, cardiopulmonary cerebral resuscitation; CRRT, continuous renal replacement therapy; Hb, hemoglobin; ref., reference range; PLT, platelet.
