# Supplementary material for: Impact of extracorporeal membrane oxygenation-related complications on in-hospital mortality
Source: PLoS One. 2024 Mar 25;19(3):e0300713. doi: 10.1371/journal.pone.0300713 (PMC10962856; doi:10.1371/journal.pone.0300713)
Supplement: S5 Table — (PDF) [file pone.0300713.s008.pdf]

S5 Table. Factors associated with ECMO-related vascular complications in VV ECMO.

	Univariable analysis<br>OR (95% CI)	*P*-value
**Age**	1.02 (0.97–1.07)	0.37
**Female**	2.99 (1.01–8.86)	0.048
**BMI**	0.98 (0.90–1.07)	0.64
**Hypertension**	2.39 (0.80–7.14)	0.12
**Diabetes mellitus**	0.84 (0.20–3.51)	0.81
**Smoking**	0.25 (0.03–1.96)	0.19
**PAOD**	9.36 (0.55–158.04)	0.12
**History of CAD**	1.49 (0.17–13.30)	0.72
**History of CVA**	4.64 (0.40–54.48)	0.22
**History of CKD**	0.00 (0.00–0.00)	<0.01
**CPCR**	0.67 (0.10–4.71)	0.69
**CRRT**	1.81 (0.62–5.25)	0.28
**ECMO running time (10 h)**	1.02 (1.01–1.04)	<0.01
**Initial Hb (ref. ≥ 10.0 g/dL)**		0.048
<8.0 g/dL	4.93 (1.21–20.07)	0.03
8.0–10.0 g/dL	0.89 (0.23–3.47)	0.87
**Initial PLT (ref. ≥ 100×10^3/μL)**	Not calculable	
<50×10^3/μL		
50–100(×10^3)/μL		

ECMO, extracorporeal membrane oxygenation; VV, venovenous; OR, odds ratio; CI, confidence interval; BMI, body mass index; PAOD, peripheral arterial occlusive disease; CAD, coronary artery disease; CVA, cerebrovascular accident; CKD, chronic kidney disease; CPCR, cardiopulmonary cerebral resuscitation; CRRT, continuous renal replacement therapy; Hb, hemoglobin; ref., reference range; PLT, platelet.
