# Supplementary material for: Impact of extracorporeal membrane oxygenation-related complications on in-hospital mortality
Source: PLoS One. 2024 Mar 25;19(3):e0300713. doi: 10.1371/journal.pone.0300713 (PMC10962856; doi:10.1371/journal.pone.0300713)
Supplement: S6 Table — (PDF) [file pone.0300713.s009.pdf]

S6 Table. Factors associated with ECMO-related cerebrovascular complications in VV ECMO.

	**Univariable analysis** **OR (95% CI)**	***P*-value**
**Age**	1.01 (0.95–1.08)	0.73
**Female**	0.51 (0.13–2.01)	0.34
**BMI**	0.93 (0.78–1.10)	0.37
**Hypertension**	2.03 (0.62–6.68)	0.24
**Diabetes mellitus**	3.80 (1.10–13.15)	0.04
**Smoking**	0.31 (0.04–2.49)	0.27
**PAOD**	4.93 (0.85–28.70)	<0.01
**History of CAD**	4.93 (0.85–28.70)	0.08
**History of CVA**	5.72 (0.48–68.20)	0.17
**History of CKD**	7.59 (1.87–30.75)	<0.01
**CPCR**	0.00 (0.00–0.00)	<0.01
**CRRT**	2.94 (0.86–9.89)	0.08
**ECMO running time (10 h)**	1.00 (0.99–1.02)	0.74
**Initial Hb (ref. ≥ 10.0 g/dL)**	Not calculable	
<8.0 g/dL		
8.0–10.0 g/dL		
**Initial PLT (ref. ≥ 100×10^3/µL)**		0.13
<50×10^3/µL	4.51 (0.71–28.73)	0.11
50–100(×10^3)/µL	3.43 (0.75–15.80)	0.11

ECMO, extracorporeal membrane oxygenation; VA, venoarterial; OR, odds ratio; CI, confidence interval; BMI, body mass index; PAOD, peripheral arterial occlusive disease; CAD, coronary artery disease; CVA, cerebrovascular accident; CKD, chronic kidney disease; CPCR, cardiopulmonary cerebral resuscitation; CRRT, continuous renal replacement therapy; Hb, hemoglobin; ref., reference range; PLT, platelet.
